# Reduced Collateral Tissue Damage Using Thermal-Feedback-Based Power Adaptation of an Electrosurgery Inverter

**DOI:** 10.1109/tpel.2022.3179301

**Published:** 2022-05-31

**Authors:** Congbo Bao, Sudip K. Mazumder

**Affiliations:** Electrical and Computer Engineering, University of Illinois at Chicago, Chicago, IL 60607 USA.

**Keywords:** Adaptation, collateral damage, electrosurgery, feedback, inverter, power, temperature, thermal

## Abstract

Well-selected power with accurate delivery is of importance in electrosurgery to generate proper temperature at the cutting site, and thus, reduce undesired collateral tissue damages. Conventional electrosurgery generator (ESG) targets tracking a preset power, manually set by surgeons per their experience before the surgery, with high accurate delivery. It is possible that this fixed power setting is not at the optimal point and, thus, increases the possibility of added-collateral biomedical tissue damage. To eliminate the potential negative impact of the fixed and ill-suited power setting, a real-time feedback control scheme is outlined in this article to adjust the preset power of the ESG to create an adaptive power reference, which is then tracked using an experimental high-frequency inverter (HFI) that enables electrosurgery with a fundamental (sinusoidal) output frequency of 390 kHz. Subsequently, experiments using the gallium nitride (GaN)-based HFI are carried out to demonstrate the efficacy of the new variable-power approach over the conventional fixed power approach in terms of collateral tissue damage reduction.

## I. Introduction

Electrosurgery has been used for around a century [1]. It applies HF voltage across conductive biomedical tissue along with the current dictated by the tissue impedance to elicit a clinical effect, such as cutting and coagulation [2]. The mechanism of biotissue incision or removal roots in the Joule energy converted from the applied electrical active power. The tissue liquid is rapidly heated up by the energy to the point of vaporization and then the tissue disperses in form of smoke and stream [1], [3]. The cutting effect on a certain tissue is tightly related to the total energy delivered to it. As is known, energy is the integration of power and time, therefore, both cutting speed and cutting power impose an impact on the final cutting effect [1]–[5]. Mismatched cutting speeding or poorly-regulated cutting power either is not able to generate desired clinical effect or may cause undesired added tissue damage, such as charring, thermal spread, dragging, and so on [4]–[6]. ESG cutting speed is exclusively controlled by surgeons according to their clinical experience and expertise. Therefore, traditional ESG aims at delivering the power, manually set by surgeons before the surgery, as accurately as possible regardless of the tissue variation. It is worth mentioning that, conventionally, this power is maintained the same during the entire electrosurgery until it is manually updated by the surgeon. As a result, the interruption of the time-sensitive surgery inevitably occurs. Furthermore, El-Kebir *et al*. [5] showed that different ESG power settings have an impact on cutting effects and thermal response, but El-Kebir *et al*. [5] incorporated no thermal-feedback-based real-time power adaptation. Therefore, there is a chance that the power is not optimally orderly set and leads to increased tissue damage or undesired cutting effects. The literature [6]–[9] either focuses on improving the power tracking accuracy or pursues a prompt response to the power setting. In [10], infrared sensing is employed to record liver surface-temperature distribution under a single power setting, and it turns out that a small radial region surrounding the electric scalpel (ES) has the highest temperature during cutting. Moreover, it is reported that thermography-based sensing can be utilized to identify solar cell aging, hotspots, and partial shading faults [11]. However, thermal sensor is employed in existing work simply for temperature measurement. None of them links the thermal feedback with real-time power adaptation for electrosurgery. As such, ill-suited power setting issue persists and its resolution via power adaptation is of interest for an ESG.

**Fig. 1.**
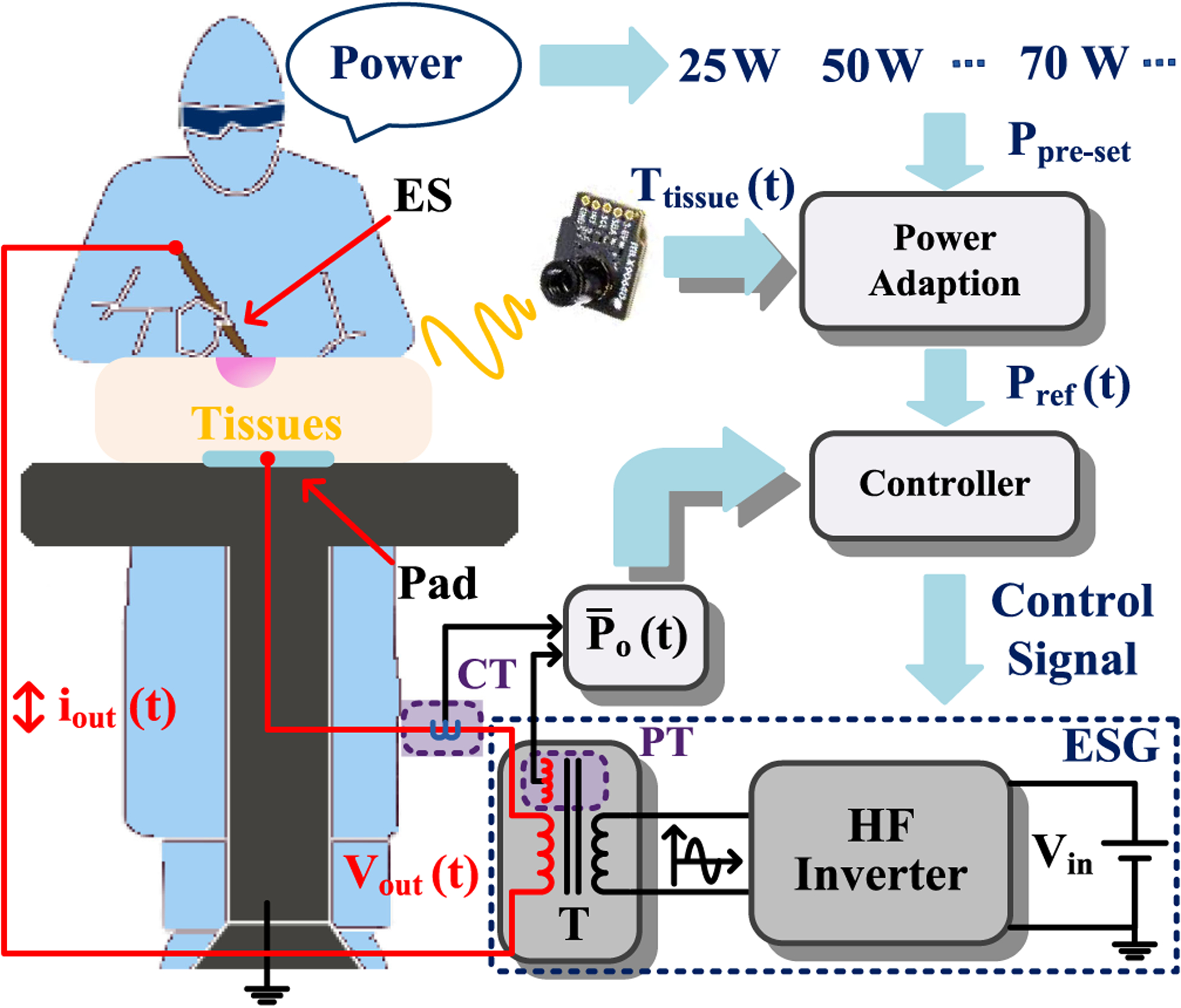
Illustration of a thermal-feedback-based monopolar electrosurgery using an ESG. The current- and voltage-sensing transformers are denoted as CT and PT, respectively. The HF transformer (*T*) boosts the output voltage (*V*_out_ (*t*)). The tissue is placed between the ES and return pad. The thermal camera monitors tissue surface temperature.

To tackle the ill-suited power setting challenge and reduce collateral tissue damage, a novel real-time thermal-feedback-based closed-loop control scheme is pursued, as illustrated in Fig. 1, in which a tissue surface temperature measurement from the electrosurgery incision site is used to adjust the ESG preset power to create an adaptive power reference, which is then tracked by a power-controller in an experimental HFI. Section II outlines how an ill-suited ESG setting can cause added tissue damage while Section III outlines the thermal-feedback-based power adaptation scheme. Section IV provides experimental results while Section V concludes this article.

## II. Illustration of Impact of Ill-Suited Power Setting

More than 70% weight of soft tissue comprises water and the tissue is removed when the applied energy vaporizes the water at 100 °C. Tissue vaporization is accompanied by another phenomenon, namely, tissue denaturation when tissue cell temperature is between 60 and 100 °C [12]. Physical properties of tissues among individuals exhibit differences associated with gender, age, size, etc. [10], and those differences escalate the extent of tissue denaturation when the constant power is indiscriminately applied. Tissue denaturation occurs due to undesired thermal spread, and it should be minimized by adjustment in power reference *P*_ref_(*t*), by modulating it with Δ*P*(*t*) around the *P*_preset_, when cutting speeding is fixed [4].

**Fig. 2.**
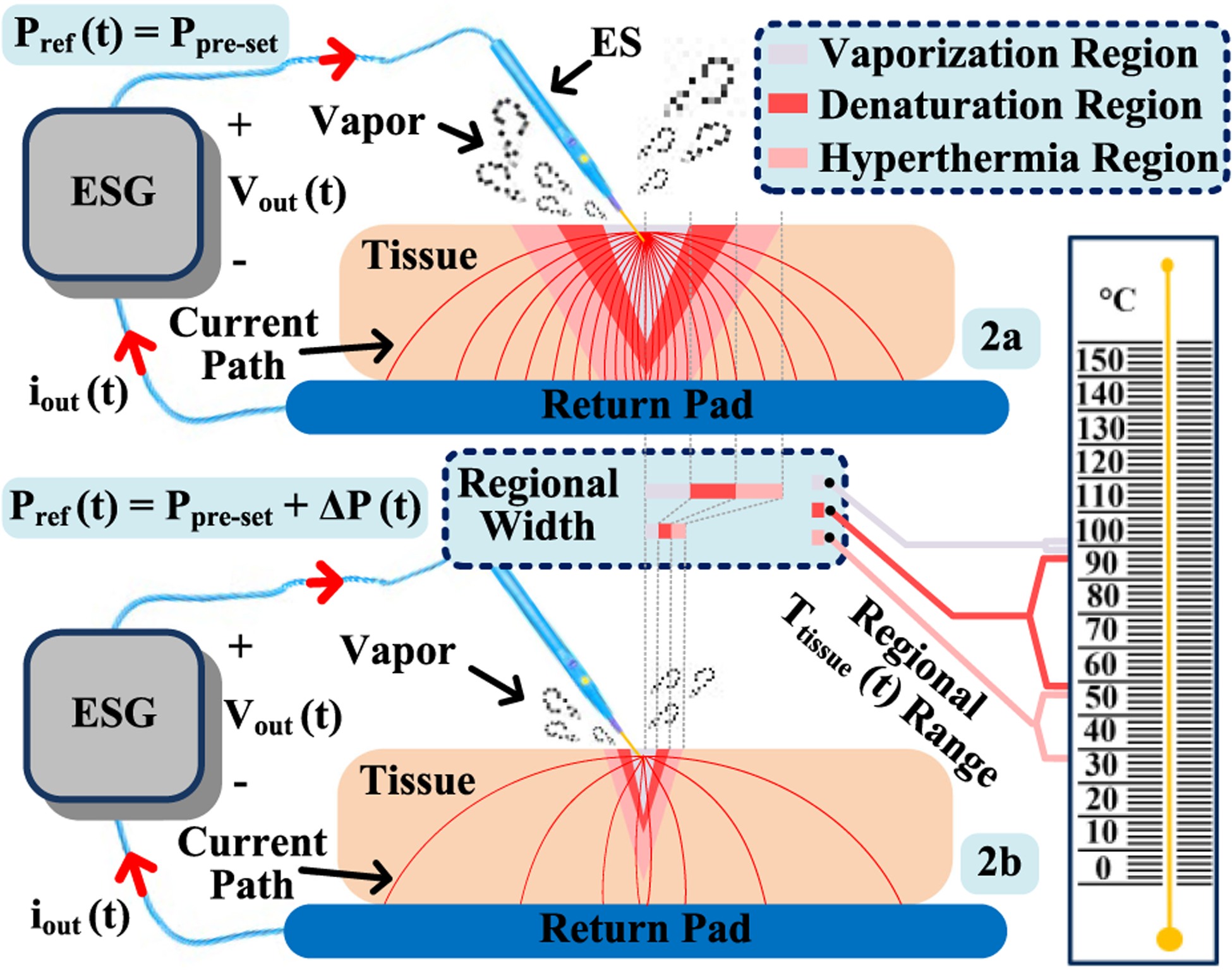
Illustration of impact of ill-suited power setting.

We illustrate this point in Fig. 2 with two scenarios. In the first scenario, as illustrated in Fig. 2(a), *P*_ref_(*t*) is not varied but kept fixed at *P*_ref_(*t*) = *P*_preset_. Because Δ*P*(*t*) = 0 for this scenario, if *P*_preset_ is ill-suited and say higher than what is really needed for safe electrosurgery, then, in the absence of power adaptation, more energy is delivered in a time interval. Consequently, the temperature of a larger volume of tissues reaches the vaporization and denaturation range, dictated by the overall specific heat capacity. As a result, unnecessary tissue removal and denaturation occur, leading to collateral damage even in the presence of a power-control loop since the latter will only ensure that ${\bar{P}}_{o}(t)=P_{\text{ref}}(t)=P_{\text{preset }}$. In the second scenario, as illustrated in Fig. 2(b), *P*_ref_(*t*) has the ability to vary with time due to the power adaptation Δ*P*(*t*) that is guided by the thermal feedback from the incision site. As such, *P*_ref_(*t*) = *P*_preset_ + Δ*P*(*t*), which ensures that a desired *P*_ref_(*t*) is obtained to minimize the collateral tissue damage.

## III. Power Control Schemes: Constant Versus Adaptive

**Fig. 3.**
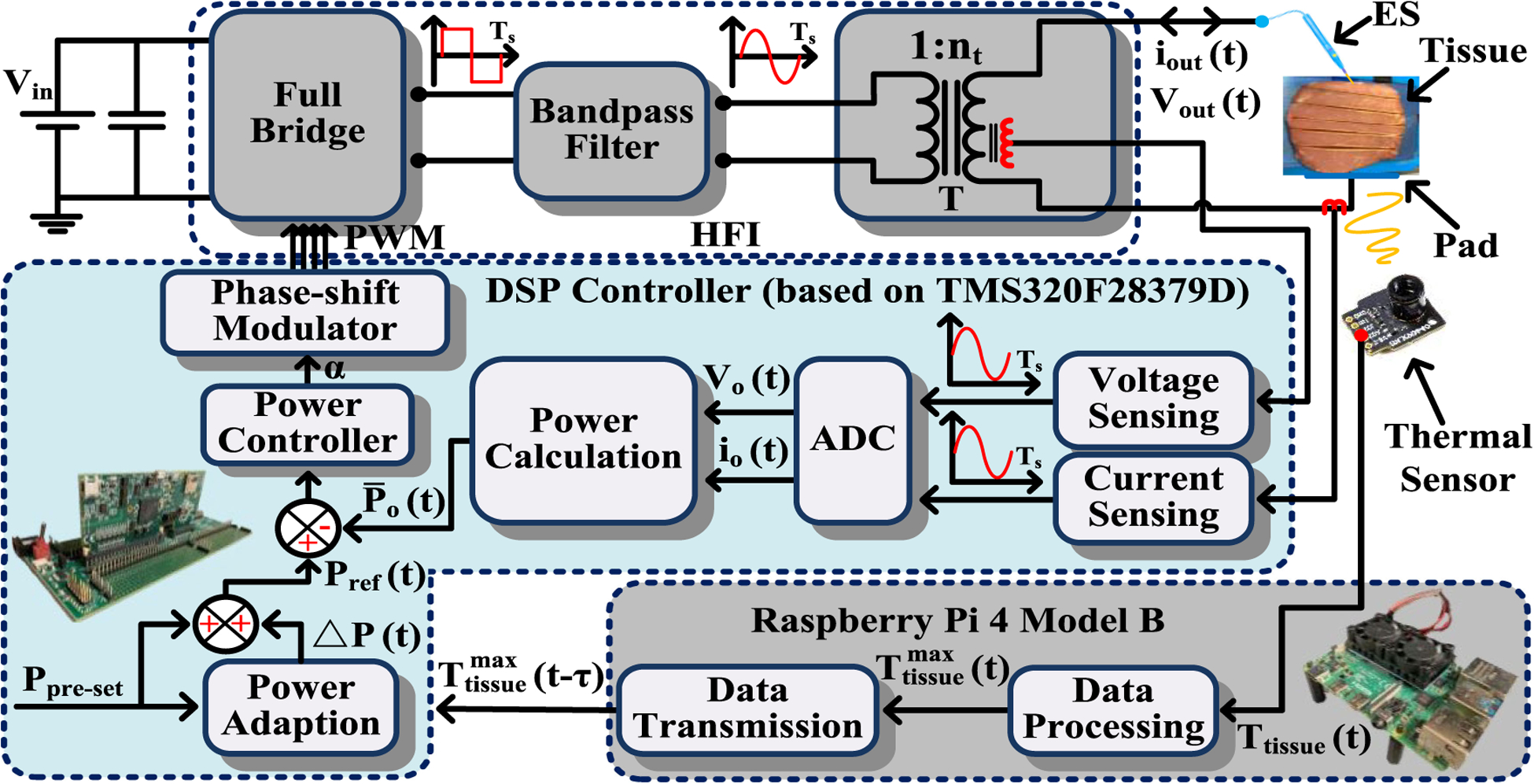
Illustration of output power control for the HFI. The power controller tracks the power reference (***P***_**ref**_ (***t***)). The thermal-feedback-based power adaption control autonomously modifies the preset power (***P***_**preset**_) via a power adaption (Δ***P***(***t***)). ADC is the analog to digital converter module. Digital signal processor (DSP) used here is TMS320F28379D. Thermal sensor is of 24 *×* 32 pixels. ***T***_**tissue**_ (***t***) is an array of dimension 24 × 32 that represents the spatio-temporally sensed tissue surface temperature while $T_{\text{tissue }}^{\text{max}}\left(t\right)$ is the maximum of ***T***_**tissue**_ (***t***) in each sensing refresh frame. Data transmission delay (*τ*) is negligible.

The full-bridge and bandpass filter-based HFI was initially introduced in [7] and detailed in [8] and it is redrawn in Fig. 3 with the integration of thermal-feedback-based power adaption.

The fundamental (sinusoidal) output frequency and switching frequency of the HFI are set to be 390 kHz. Referring to Fig. 3, the ideal sensed output voltage of the abovementioned HFI (*V*_*o*_(*t*)) is captured

(1) $$V_{o}(t)=\frac{4V_{\text{in}}}{\pi }\cdot n_{t}\cdot \text{cos}(\alpha )\cdot \text{sin}\;\left(2\pi f_{s}t\right)\cdot v_{\text{scaling }}$$

where *V*_in_and *f*_*s*_ are the input voltage and the full-bridge switching frequency, respectively. *n*_*t*_ is the transformer turn ratio, *α* is the phase shift angle between the diagonal switches in the full-bridge [8], and *v*_scaling_ represents the voltage-sensor scaling. The corresponding output power *P*_*o*_(*t*) for a linear load, with a load angle of *θ*, is given by

(2) $$P_{o}(t)=\frac{V_{o_{\_}\text{pk}}\cdot I_{o_{\_}\text{pk}}}{2}\cdot \left(\text{cos}\;(\theta )-\text{cos}\;\left(4\pi f_{s}t+\theta \right)\right)\cdot \rho_{\text{scaling }}$$

where *V*_*o*_pk_ is the peak of *V*_*o*_(*t*) and *I*_*o*_pk_ is the peak of *i*_*o*_(*t*), the scaled output current of the HFI. *ρ*_scaling_ is the coefficient that maps sensed *V*_*o*_(*t*) and *i*_*o*_(*t*) back to actual output power. As seen, the first item of the *P*_*o*_(*t*) is a constant component, which is the average power $\left({\bar{P}}_{o}(t)\right)$ over an HFI fundamental-output cycle while the second term represents an ac perturbation term that evolves at double the fundamental-output frequency. This article aims to properly control the output voltage, and thus, current, such that ${\bar{P}}_{o}(t)$ is well-regulated and follows the adaptive power reference to reduce collateral tissue damage.

### A. Constant Power Control

The control block diagram in Fig. 3 achieves constant power control by setting Δ*P*(*t*) = 0 and yielding *P*_ref_(*t*) = *P*_preset_.

The constant power reference is then compared with feedback ${\bar{P}}_{o}(t)$ and the error is fed to a proportional-integral-based (PI) controller, which ensures the realization of the desired power. In actual electrosurgery, surgeons select *P*_preset_ according to their clinical needs and professional experience. However, owing to physical properties variations among patients, *P*_preset_ may be improperly set, which may cause erroneous incision-site temperature due to a mismatch between the fixed power reference set for the electrosurgery (i.e., *P*_ref_(*t*) = *P*_preset_) and the *P*_ref_(*t*) that is actually needed. Consequently, undesired cutting effects and collateral tissue damages are elicited, such as thermal spread, carbonization, and burns.

### B. Thermal-Feedback-Based Power-Adaptation Control

To mitigate the collateral tissue damage due to nonoptimality in *P*_preset_, the proposed thermal-feedback-based power adaptation control monitors tissue surface temperature using an infrared sensor and feeds it back at a frequency of 8 Hz. Based on the sensed temperature data, the power-adaptation controller feeds in real time a power adaption term (Δ*P*(*t*))

(3) $$\Delta P(t)=P_{\text{preset }}\cdot \left(\frac{T_{\text{nom }}}{\text{max}\left(T_{\text{tissue }}(t)\right)}-1\right)$$

to the *P*_ref_(*t*) yielding the following, as captured in Fig. 3

(4) $$P_{\text{ref }}(t)=P_{\text{preset }}+\Delta P(t).$$

In (3), *T*_tissue_(*t*) is the tissue surface temperature, max (*T*_tissue_(*t*)) is the maximum of *T*_tissue_(*t*), and *T*_nom_ is the nominal tissue temperature that ensures safe electrosurgery with minimal/no collateral damage. By following (4), any time the max (*T*_tissue_(*t*)) exceeds *T*_nom_, *P*_ref_(*t*) is so adjusted such that the incision-site tissue temperature is brought back close to *T*_nom_ thereby mitigating collateral tissue damage.

## IV. Experimental Results

**Fig. 4.**
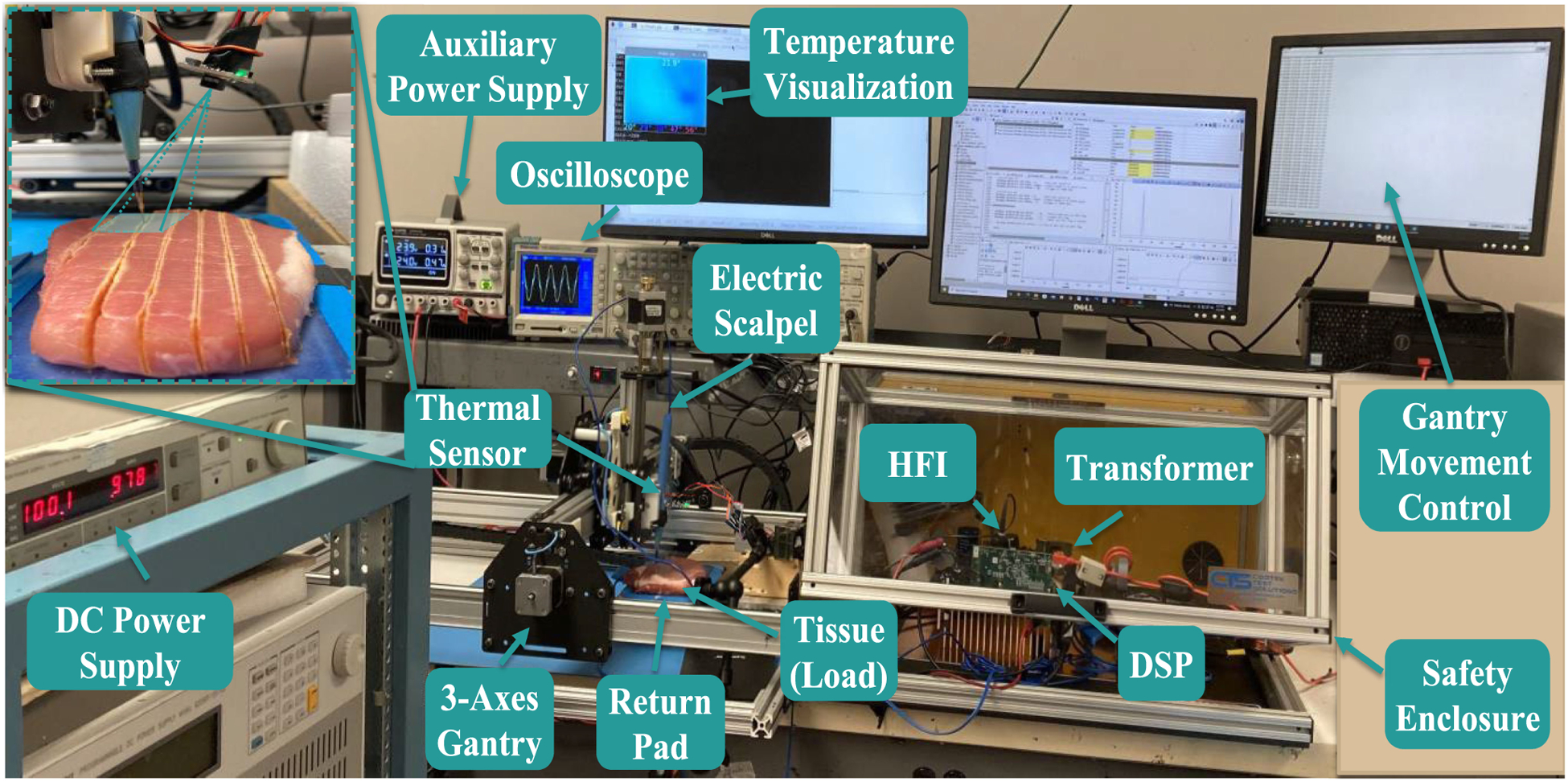
Experimental setup of the HFI. Programmable Emile3 3-axes robotic gantry is employed to hold the ES and move it in a specified direction and at a uniform speed for the repeatability of the biomedical tissue cutting experiments.

A gallium nitride field effect transistor (GaN-FET)-based hardware prototype, as shown in Fig. 4, is used to examine the cutting effects of the proposed thermal-feedback-based power adaptation control. As shown in the top left corner in Fig. 4, MLX90640 infrared sensor is mounted together with ES such that they move together without relative movement. Consequently, tissue surface temperature around ES is always monitored, and thus, the maximum temperature of entire tissue surface is captured during electrosurgery. The sensed thermal data are processed by Raspberry Pi 4 Model B and then transmitted to the DSP controller, which, using this feedback, implements the power adaptation and subsequently, generates the PWM signals for the GaN FETs (i.e., GS66508B from GaN System) of the full-bridge converter of the HFI, as shown in Fig. 3. Fresh pork with 16 millimeters (mm) thickness is placed between the ES and return pad as biomedical load of the HFI. The detailed experimental parameters are summarized in Table I.

**Fig. 5.**
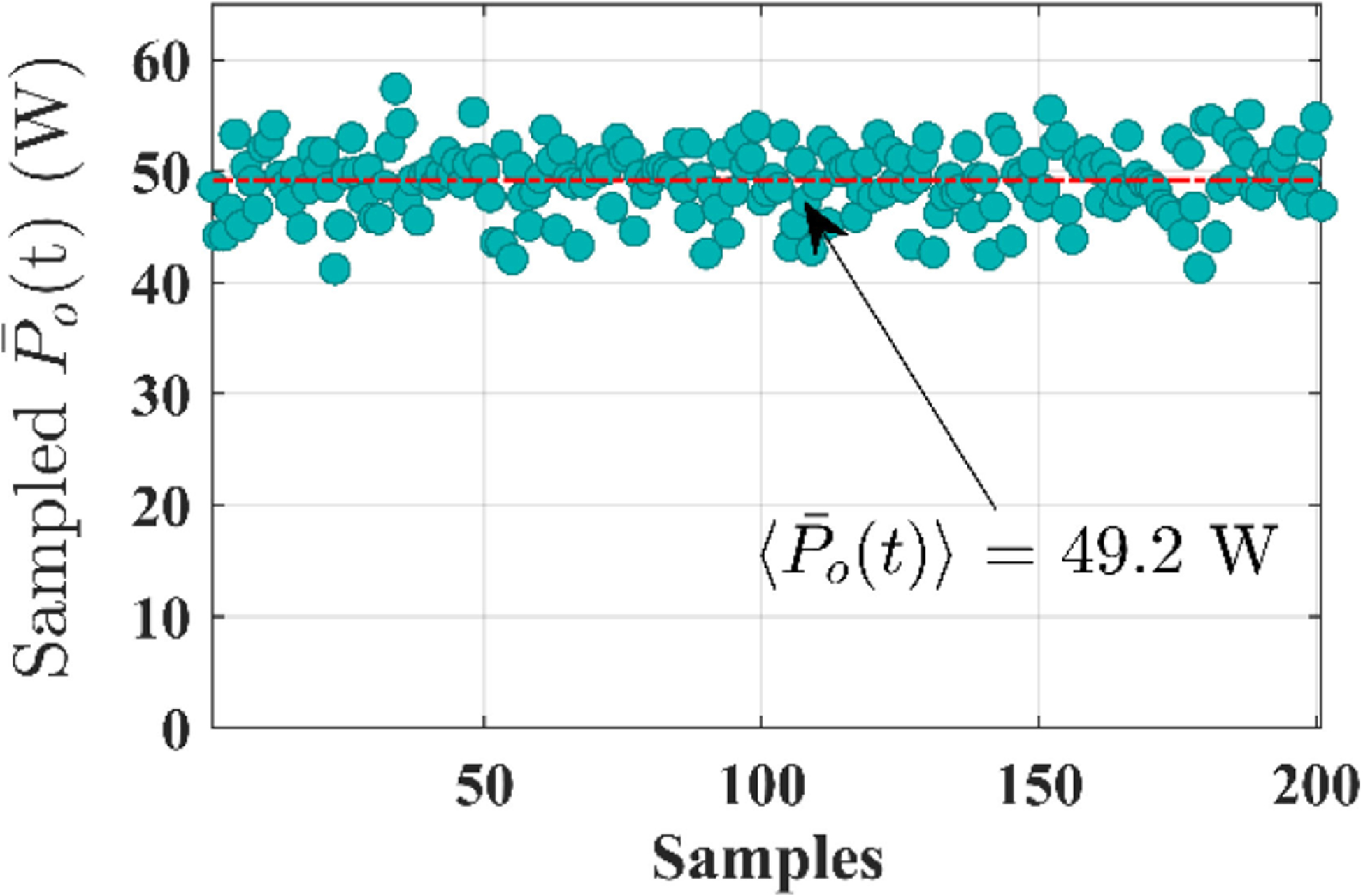
Statistical distribution of output power $\left({\bar{P}}_{o}(t)\right)$. Symbol $\langle {\bar{P}}_{o}(t)\rangle$ represents the overall average of ${\bar{P}}_{o}(t)$ but spread over all of the 200 samples.

Using this setup, first, the efficacy of HFI operating, under constant power control with *P*_ref_(*t*) = *P*_preset_ = 50 W (i.e., Δ*P*(*t*) = 0) is shown in Fig. 5 with experimental results. This scenario emulates a conventional electrosurgery, where the required output power is preset by the surgeons and the setting remains the same till the surgeons manually change it again.

**Fig. 6.**
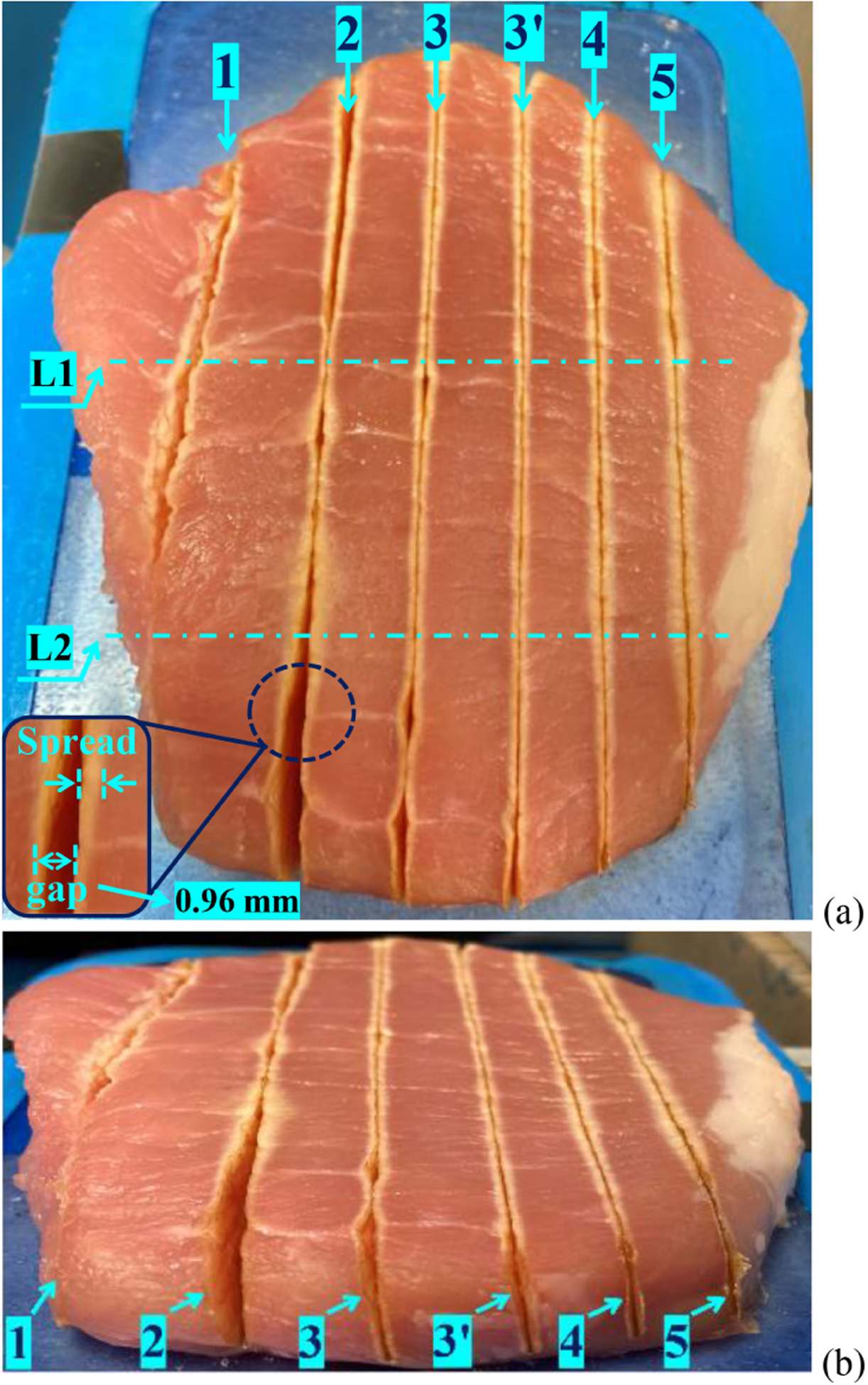
Capture of numbered test scenarios. (a) Top view. (b) Front view.

Next, in Fig. 6, the scenario when *P*_ref_(*t*) = *P*_preset_ + Δ*P*(*t*) is explored. Five test scenarios are pursued as captured in Table II: test scenarios 1 and 2 correspond to Δ*P*(*t*) = 0 and *P*_preset_ set at 50 W and 60 W, respectively; test scenarios 3–5 correspond to Δ*P*(*t*) ≠ 0 and instead, Δ*P*(*t*) is generated for each of the three scenarios based on three values of nominal temperature (*T*_nom_) of 50 °C, 55 °C, and 65 °C, respectively.

The purpose of the test scenarios 1 and 2 is to provide an illustrative approach to the choice of *T*_nom_ for determining Δ*P*(*t*) using (3) [to obtain *P*_ref_(*t*) using (4)] in the last three scenarios. As evident in Fig. 6 and Table III, the test scenario 1 corresponding to *P*_ref_(*t*) = *P*_preset_ = 50 W yields less cutting gap and thermal spread than test scenario corresponding to *P*_ref_(*t*) = *P*_preset_ = 60 W. Based on these two tests, an estimated *T*_nom_ range was conjectured to be between 50 and 65 °C guided by the outcomes of test scenarios 1 and 2.

**Fig. 7.**
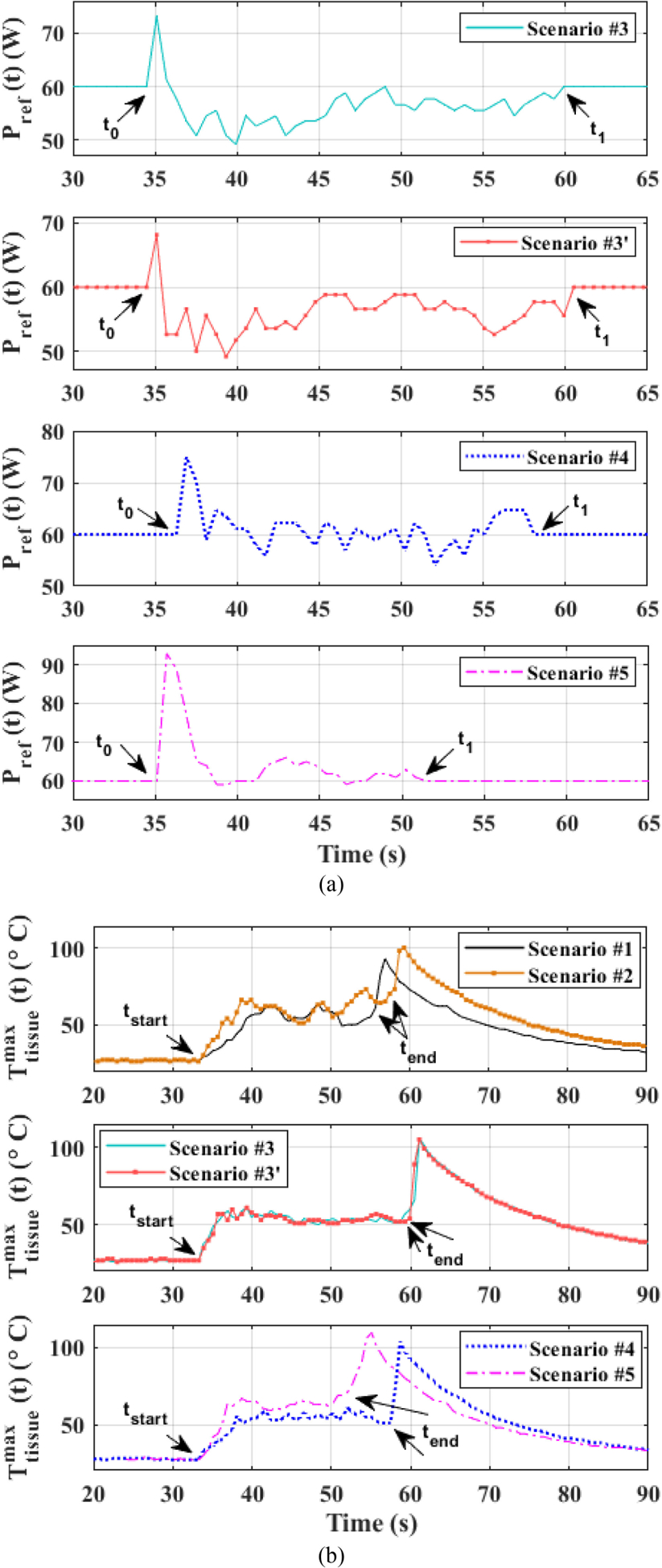
(a) Adaptive power reference that comes into effect at ***t***_0_ where the maximum tissue surface temperature ***T***_**tissue**_ (***t***) exceeds 40 °C (slightly higher than normal body temperature) and ends at ***t***_1_. (b) Maximum of sensed tissue surface temperature ***T***_**tissue**_ (***t***). Cutting period starts at ***t***_**start**_ and ends at ***t***_**end**_. The temperatures of test scenarios 1 and 2 fluctuate with a larger ripple due to lack of power adaption. However, test scenarios with power adaption, namely test scenarios 3–5, feature smaller temperature ripples and, thus, contribute to improved cutting uniformity. The hottest part of the ES is buried inside the tissue during cutting process and thermal sensor, under nominal condition, does not have access to it till the end of cutting. ES tip buried during cutting is directly exposed to thermal sensor after the cutting is completed, and thus, a temperature spike occurs in the plot, which gradually decays afterward.

Using that range, the test scenarios 3–5, following Table II, were pursued and the results are captured in Table III. The latter shows that test scenario 3 together with its repeated trial 3’ yields overall the best results and this is evident in Fig. 6 as well. It illustrates that, compared to test scenario 1, which yielded comparable cutting temperature but with a larger temperature ripple, the results are better for test scenario 3 because of the power reference adaptation, as shown in Fig. 7(a), which also adjusts the maximum tissue surface temperature accordingly with smaller temperature ripple, as shown in Fig. 7(b). The superior result for test scenario 3 over test scenarios 4 and 5, as evident in Table III and Fig. 7, is attributed to the fact that the *T*_nom_ for the latter two cases are set to be higher, leading to higher averaged cutting site temperature and thus wider cutting gap and thermal spread. It worth mentioning that the thermal spread repeatability of test scenario 3 are demonstrated by test 3’ and can be extended to other scenarios as well. Furthermore, the cutting gap uniformity with power adaption control is also repeatable, as verified by Table III.

## V. Conclusion

This article outlines a thermal-feedback-based power adaption control to reduce the collateral tissue damage for electrosurgery. The impact of the ill-suited power setting is illustrated, and the power adaption scheme is elaborated. A GaN-based HFI setup is used to examine the cutting effects of constant power and thermal-feedback-based power adaption control in terms of cutting gap and thermal spread. The experiment results show that the cutting test with a higher constant power features a larger cutting gap and wider thermal spread. Moreover, with a constant power setting, the tissue is not evenly cut with visible cutting gap and thermal spread difference at two-incision locations. With the proposed power adaption control, the cutting gap and thermal spread are tangibly reduced with the proper choice of nominal temperature. Moreover, it is also found that thermal sensor location and its resolution have an impact on the accuracy of sensing the surface temperature of the tissue and needs careful attention. Further, the gap and thermal difference at different incision sites are found to be reduced compared to results obtained using a fixed power setting. In other words, cutting uniformity with power adaption is improved in terms of both cutting gap and thermal spread. It is evident that power adaptation in the vicinity of accurate nominal temperature is the key to reduced collateral damage. In practical electrosurgery, this estimate can be obtained more accurately given the repeatability of reliable cutting performance and the availability of a much larger electrosurgery database.

## Figures and Tables

**TABLE I:** Parameters for the Experimental Hardware Setup

Parameters	Values
Input voltage (*V*_*in*_)	110 V
Scaling factor	*v*_scaling_ = 0.00415, *ρ*_scaling_ = 154.60
HFI parameters	The same as in [8]
ES moving speed	5 mm/s
PI parameters	*K*_*p*_ = 0.035, *K*_*i*_ = 160 000

**TABLE II:** Operating Conditions for the Five Test Scenarios

_Settings_╲^Trials^	Test Scenarios					
	#1	#2	#3	#3′	#4	#5
*P*_preset_	50 W	60 W	60 W	60 W	60 W	60 W
Δ*P*(*t*)	0	0	Using (3), *T*_nom_ = 50 °C	Using (3), *T*_nom_ = 50 °C	Using (3), *T*_nom_ = 55 °C	Using (3), *T*_nom_ = 65 °C
*P*_ref_ (*t*)	50 W	60 W	Using (4)	Using (4)	Using (4)	Using (4)

**TABLE III:** Evaluation of Experimental Cutting Effects for the Five Test Scenarios

_Metrics_╲^Trials^	Test Scenarios					
	#1	#2	#3	#3′	#4	#5
Gap at *L*1	0.66	0.06	0.10	0.04	0.09	0.06
Gap at *L*2	0.02	0.40	0.05	0.05	0.10	0.19
Spread *L*1	0.90	0.82	0.95	0.98	1.37	1.51
Spread *L*2	0.02	1.99	0.99	0.90	1.16	1.70
Gap difference	0.64	0.34	0.05	0.01	0.01	0.13
Spread difference	0.88	1.17	0.04	0.08	0.21	0.19

Note: All measurements are gauged in mm.
